# Blood Proteomics Analysis Reveals Potential Biomarkers and Convergent Dysregulated Pathways in Autism Spectrum Disorder: A Pilot Study

**DOI:** 10.3390/ijms24087443

**Published:** 2023-04-18

**Authors:** Areej Mesleh, Hanan Ehtewish, Alberto de la Fuente, Hawra Al-shamari, Iman Ghazal, Fatema Al-Faraj, Fouad Al-Shaban, Houari B. Abdesselem, Mohamed Emara, Nehad M. Alajez, Abdelilah Arredouani, Julie Decock, Omar Albagha, Lawrence W. Stanton, Sara A. Abdulla, Omar M. A. El-Agnaf

**Affiliations:** 1 College of Health and Life Sciences (CHLS), Hamad Bin Khalifa University (HBKU), Qatar Foundation (QF), Doha P.O. Box 34110, Qatar; 2 Neurological Disorders Research Center, Qatar Biomedical Research Institute (QBRI), Hamad Bin Khalifa University (HBKU), Qatar Foundation (QF), Doha P.O. Box 34110, Qatar; 3 Diabetes Research Center, Qatar Biomedical Research Institute (QBRI), Hamad Bin Khalifa University (HBKU), Doha P.O. Box 34110, Qatar; 4 Proteomics Core Facility, Qatar Biomedical Research Institute (QBRI), Hamad Bin Khalifa University (HBKU), Qatar Foundation (QF), Doha P.O. Box 34110, Qatar; 5 Basic Medical Sciences Department, College of Medicine, QU Health, Qatar University (QU), Doha P.O. Box 2713, Qatar; 6 Translational Cancer and Immunity Center, Qatar Biomedical Research Institute (QBRI), Hamad Bin Khalifa University (HBKU), Qatar Foundation (QF), Doha P.O. Box 34110, Qatar

**Keywords:** ASD, autism, biomarkers, early diagnosis, PEA, proteomics, blood profiling, machine learning, patient stratification

## Abstract

Autism spectrum disorder (ASD) is an umbrella term that encompasses several disabling neurodevelopmental conditions. These conditions are characterized by impaired manifestation in social and communication skills with repetitive and restrictive behaviors or interests. Thus far, there are no approved biomarkers for ASD screening and diagnosis; also, the current diagnosis depends heavily on a physician’s assessment and family’s awareness of ASD symptoms. Identifying blood proteomic biomarkers and performing deep blood proteome profiling could highlight common underlying dysfunctions between cases of ASD, given its heterogeneous nature, thus laying the foundation for large-scale blood-based biomarker discovery studies. This study measured the expression of 1196 serum proteins using proximity extension assay (PEA) technology. The screened serum samples included ASD cases (n = 91) and healthy controls (n = 30) between 6 and 15 years of age. Our findings revealed 251 differentially expressed proteins between ASD and healthy controls, of which 237 proteins were significantly upregulated and 14 proteins were significantly downregulated. Machine learning analysis identified 15 proteins that could be biomarkers for ASD with an area under the curve (AUC) = 0.876 using support vector machine (SVM). Gene Ontology (GO) analysis of the top differentially expressed proteins (TopDE) and weighted gene co-expression analysis (WGCNA) revealed dysregulation of SNARE vesicular transport and ErbB pathways in ASD cases. Furthermore, correlation analysis showed that proteins from those pathways correlate with ASD severity. Further validation and verification of the identified biomarkers and pathways are warranted.

## 1. Introduction

Autism spectrum disorder (ASD) is an umbrella term that encompasses various disabling neurodevelopmental conditions. These conditions are characterized by impaired manifestation in social and communication skills with repetitive and restrictive behaviors or interests [1]. The *Diagnostic and Statistical Manual of Mental Disorders, Fifth Edition* (DSM-5), described ASD as a continuum of symptoms that ranges from mild to severe in the abovementioned domains [1]. Furthermore, it is not uncommon for ASD individuals to suffer from other comorbidities such as intellectual disability, attention-deficit/hyperactivity disorder, epilepsy, gastrointestinal disturbances, and psychiatric disorders such as anxiety and depression [2,3,4]. According to the autism and developmental disabilities monitoring network surveillance, the prevalence of ASD has been increasing in the past two decades [5]. The estimated global prevalence of ASD is 0.6–1.7% [6], and in the United States, ASD cases are reported in 1 in every 44 children [7]. A cross-sectional study published in 2019 found that the prevalence of ASD in Qatar is 1.14% amongst 5–12-year-old children [8]. It is still uncertain why the prevalence of ASD is increasing; however, the continuous change in the diagnostic criteria may impact the prevalence [9]. ASD is a heritable disorder, although it presents heterogenicity and complexity in terms of genetics and clinical manifestation. A study has shown that monozygotic and dizygotic twins have 60–92% and 0–10% ASD concordance rates, respectively [10]. Paradoxically, only 30–40% of ASD cases are related to genetic causes, leaving the majority of the cases labeled as idiopathic ASD [11]. Furthermore, some studies point toward environmental predispositions such as parental age, birth injuries, viral infection, exposure to toxins and drugs, and maternal autoimmunity as potential risk factors for ASD [12,13,14,15]. Thus far, there is no approved biomarker for ASD screening and diagnosis, and the current diagnosis depends heavily on a physician’s assessment and family’s awareness of ASD symptoms. A late positive diagnosis of ASD after an initial negative diagnosis is not uncommon [16]. Moreover, there is a possibility of gender bias that results in females being underdiagnosed, as females are less likely to present with overt ASD symptoms [17]. As a result, having a set of biomarkers that mirror the underlying pathophysiology of ASD is needed to support the current diagnostic methods and help untangle the complexity of ASD. In addition, identifying biomarkers for early diagnosis is crucial for implementing early behavioral interventions and thus improving ASD outcomes [18]. The genomic architecture of ASD has been widely studied in different populations [19,20,21], and more recently, there has been an emerging interest in studying the ASD blood proteome using different assays such as label-free mass spectrometry, immunoassays, and aptamer-based assays [22,23,24]. Nonetheless, more studies are needed in this realm, especially in the Middle Eastern population as it has been underrepresented. This study has two main aims: first, to identify an oasis of potential blood proteomic biomarkers of ASD in the Qatari population; second, to perform deep blood proteome profiling discovery that could highlight common underlying dysfunctions between cases of ASD, given its heterogeneous nature. Therefore, this pilot study utilizes the serum of children with ASD and healthy controls in an effort to identify blood biomarkers for ASD and characterize molecular pathways potentially involved in ASD pathogenesis.

## 2. Results

### 2.1. Study Cohort Characteristics

**Figure 1:**
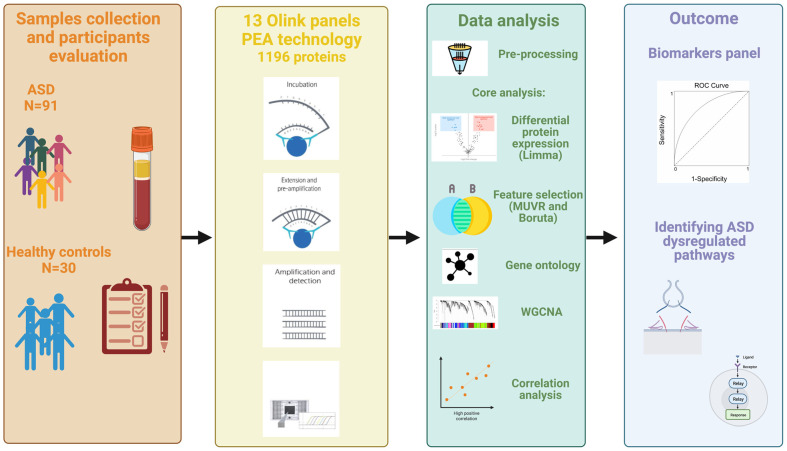
Study overview. Serum samples from 91 ASD cases and 30 healthy controls (HCs) were collected along with the participants’ demographical information. The serum samples were analyzed using 13 Olink target panels. Different bioinformatic and statistical analysis approaches were deployed for blood proteome profiling to identify potential biomarkers for ASD diagnosis and dysregulated pathways. (Created with www.BioRender.com (accessed on 18 March 2023).)

An overview of the study design is illustrated in Figure 1. This study included a total of 121 participants, 91 ASD cases and 30 healthy controls (HCs) that ranged from 6 to 15 years of age. The average ages for ASD cases and HCs were 8.32 ± 2.29 and 11.1 ± 2.2, respectively. The male-to-female ratio in the HC group was equal (50%); however, the majority of ASD cases (79%) were males due to the high prevalence of ASD in males, which can reach up to 4:1 [25]. All ASD cases were clinically diagnosed with ASD using DSM-5. Subsequently, the severity level of 50 ASD cases was assessed using the ADOS-2 score. The demographical information of ASD cases and HCs is summarized in Table 1.

### 2.2. ASD Proteome Profiling Reveals Altered Protein Expression in ASD Individuals Compared to HCs

**Figure 2:**
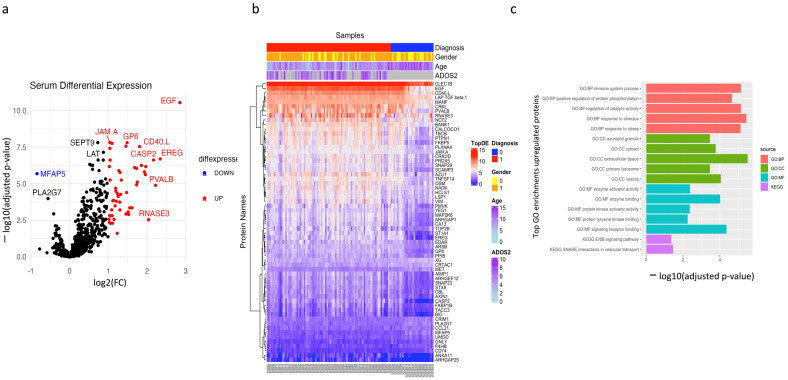
Differential expression of ASD blood proteome. (**a**) The volcano plot shows log2 fold change (*x*-axis) against Limma −log10 adjusted *p*-value (*y*-axis) for all the proteins expressed between HCs and ASD cases. Top upregulated and downregulated proteins with an FC > 2 are labeled in red and blue, respectively. (**b**) Supervised heatmap of ASD cases and HCs using the top differentially expressed proteins (TopDE, n = 54), gender (male = 1, female = 0), and diagnosis (ASD = 1, HCs = 0). (**c**) Top GO terms of the upregulated proteins, from TopDE list.

The main aim of this study is to identify differentially expressed proteins in peripheral blood between ASD cases and HCs. These proteins could be used for proteomics blood profiling, pathway enrichment analysis, and biomarker discovery. A total of 1124 proteins passed the quality control measures and were further used for downstream analysis. Similarly, sample outliers were removed prior to the downstream analysis, as shown in Figure S1. Differentially expressed proteins were identified using *Limma* package in R, and the model was adjusted for covariates (age and sex). A total of 251 proteins were significantly differentially expressed between ASD cases and HCs (BH-adjusted *p*-value < 0.05), of which 237 were upregulated and 14 were downregulated. To establish a list of the top differential expression (TopDE), a protein was listed if it fulfilled the following criteria: (a) it exhibited a BH-adjusted *p*-value < 0.05, (b) it showed a minimum of 2-fold difference between ASD cases and HCs. As illustrated in Figure 2a, a total of 53 upregulated proteins and one downregulated protein were listed in the TopDE. The results of the TopDE proteins are depicted in a supervised heatmap (Figure 2b). Further details of all the significantly expressed proteins are listed in Table S1a,b.

### 2.3. Gene Ontology Enrichment Analysis of the Differentially Expressed Proteins

Gene Ontology (GO) enrichment analysis of the top upregulated proteins (n = 53) indicated an overrepresentation of various biological processes that include response to stress, immune response, signal transduction, protein phosphorylation, cell–cell communication, apoptosis, and vesicular transport. Cell components showed an overrepresentation of a wide range of intra- and extracellular vesicles, as well as sorting endosomes and exosomes. KEGG database analysis showed enrichment in proteins associated with SNARE interactions in vesicular transport and the ErbB signaling pathway. Furthermore, REACTOME database analysis showed an overrepresentation of various signaling pathways such as signaling by non-receptor tyrosine kinase, signaling by PTK6, regulation of signaling by CBL, and signaling by EGFR; these pathways share the following proteins: EGF, EREG, CBL, and PTPN1. Since there is only one downregulated protein (MFAP5) in the TopDE list, we did not perform GO on that protein; however, it is known that MFAP5 is involved in immune system processes and localized extracellularly [26]. A summary of the top GO terms in the upregulated list is shown in Figure 2c and Table S2.

### 2.4. Machine Learning Identifies Potential Biomarkers for ASD Diagnosis

To demonstrate that the proteins in the TopDE list can be used as biomarkers for predicting the diagnosis, we employed machine learning algorithms. We first performed variable selection to obtain a short list of the best predictors and then evaluated this biomarker signature using a variety of classification approaches to distinguish between ASD cases and healthy controls (for further information, refer to Section 4.6).

**Figure 3:**
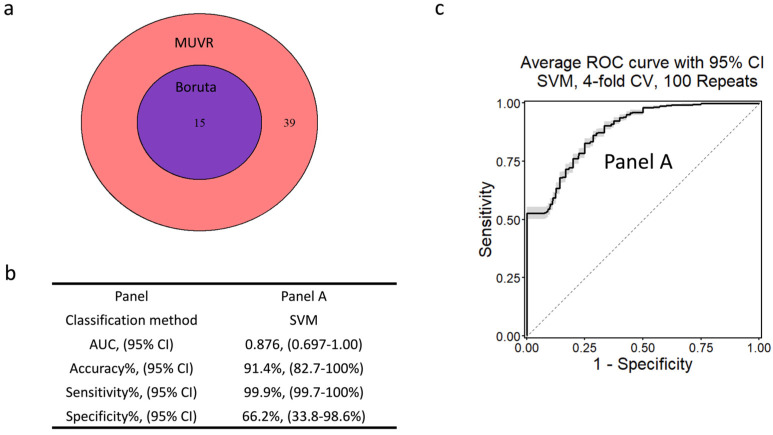
Machine learning outcome. (**a**) A Venn diagram of the overlapping proteins between MUVR and Boruta, feature selection algorithms. (**b**) The performance characteristics of the best classification method (SVM). (**c**) An ROC curve of Panel A’s SVM outcome showing the trade-off between true positive rate (sensitivity) and false positive rate (1 – specificity) for different classification thresholds.

We applied minimal unbiased variable selection (MUVR) [27] and Boruta [28] for feature selection using the TopDE list; the list of the TopDE proteins resembled a univariant pre-filter for the dataset. MUVR selected all proteins in the TopDE list for optimal classification (Figure S1a). Furthermore, Boruta selected only 15 proteins as the best predictors for the diagnosis. To minimize the number of biomarkers and to increase our confidence, we selected the overlapping proteins between MUVR and Boruta. As shown in Figure 3a, 15 proteins overlapped between MUVR and Boruta. Interestingly, these 15 overlapping proteins were the top-ranked proteins in the MUVR list (most informative for differentiating ASD cases from HCs). Details of these 15 proteins are listed in Table 2, and these proteins will be referred to as “Panel A”. Subsequently, the diagnostic performance of Panel A was tested using multiple multivariant supervised machine learning algorithms (random forest, generalized linear model-net, and support vector machine (SVM)). The Panel A dataset was internally validated with four-fold cross-validations and 250 repeats. The result of the machine learning showed that, overall, SVM slightly outperformed RF and generalized linear model-net models (see Figure S1b,c). Panel A showed the following performance characteristics: AUC = 0.876, accuracy = 91.4%, sensitivity = 99.9%, specificity = 66.2% (Figure 3b). The receiver operating characteristic (ROC) curve of Panel A is shown in Figure 3c. Therefore, Panel A proteins can be used as potential diagnostic biomarkers for ASD.

### 2.5. Proteome Co-Expression Network Analysis Uncovers Modules That Correlate with ASD Clinical Traits

**Figure 4:**
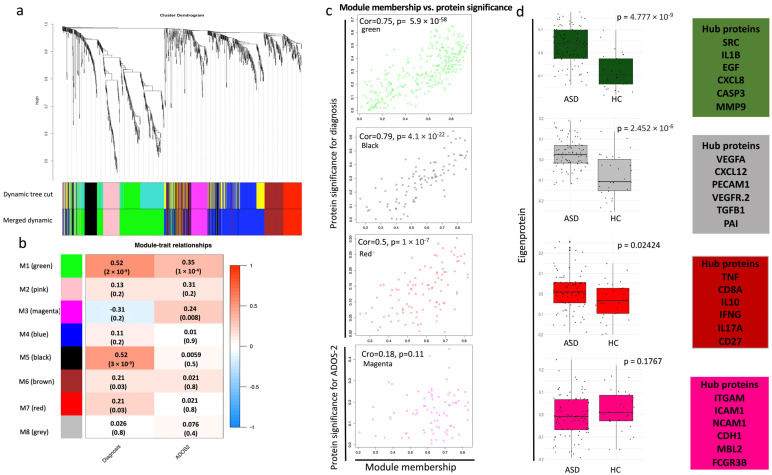
Weighted gene co-expression network analysis (WGCNA). (**a**) Protein dendrogram obtained by average linkage hierarchical clustering of the 1124 proteins. The color bands underneath the dendrogram show the module obtained from the dynamic tree cut. (**b**) The module trait relationship (*p*-value and correlation) for the identified modules in relation to the clinical traits. (**c**) Scatterplots of protein significance versus module membership in modules that significantly correlated with the clinical phenotypes (M1, M3, M5, M7). Protein significance versus module membership exhibits a significant correlation. Module membership is each protein’s expression level correlated with the module’s eigengene/epiprotein (the first principal component). For a protein to be assigned to a particular module, its module membership value must be close to 1 or −1. (**d**) Boxplots of the eigenprotein (the first principal component value of each sample) between ASD cases and HCs in the significant modules, and the boxes on the right show the hub proteins in each module; *t*-test was used to assess the significance between the two groups (*p*-value < 0.05).

Weighted gene co-expression network analysis (WGCNA) was conducted on all the proteins. This algorithm divides the proteins into different modules based on their co-expression pattern [29,30]. Thus, we constructed a co-expression model using all ASD cases and HCs; eight modules were generated with different numbers of proteins and various levels of correlation with the diagnostic status, ASD versus HC, and the ADOS-2 score. These modules are M1 green, M2 pink, M3 magenta, M4 blue, M5 black, M6 brown, M7 red, and M8 grey (Figure 4a, Table S3). Three modules showed a significant correlation with the diagnostic status (M1, M5, and M7) (Figure 4b). The magenta module (M3) showed a significant correlation with the ADOS-2 score (Figure 4b). A scatter plot of the protein significance versus the module membership of M1, M3, M5, and M7 (the modules that correlated with the clinical traits) exhibited a significant correlation (Figure 4c); however, the magenta M3 module did not show a high level of significance.

To investigate the functions of the significantly correlated modules, GO enrichment analysis was performed for each module (data not shown). Interestingly, the results showed that the green M1 module, which harbors the majority of TopDE proteins, has a similar GO enrichment to that of the TopDE list. It included SNARE vesicular transport, ErbB signaling pathways (as well as other signaling pathways), immune cell activation, and inflammatory markers. Importantly, the green module showed further overrepresentation of neural-related ontologies such as neuronal death, FOX-mediated transcription of neuronal genes, NAD+ metabolism, and cell leading edge, a biological process involved in the growth of axons and dendrites. Six proteins from this module are from the TopDE list (CASP2, PLXNA4, CBL, BID, NMNAT1, and NADK). Furthermore, several developmental process themes such as tub, embryo, vasculature, and epithelial development, are enriched in M1. Although the black M5 module showed a similar overall pattern to M1 GO enrichment, it showed overrepresentation in cerebellum proteins in Human Proteome Atlas (HPA) category. The black M5 module exhibited enrichment in other neuronal-related processes, such as neuregulin and cellular response to beta-amyloid, as well as the activation of matrix metalloproteinases, which plays a crucial role in neuroinflammation and brain development [31]. In contrast, the red M7 module was dominated by immune-related terms such as cytokine–cytokine receptor interaction; interleukin signaling (i.e., IL-17 and IL-10); inflammation; and decreased production of antibodies, autoimmunity, and immune cell proliferation and migration.

Unlike M1, M5, and M7 modules, magenta M3 is enriched with semaphorin receptor activity. Some of the significantly downregulated proteins (MET and PLXNB2) that were co-expressed in this module play a crucial role in semaphorin function. Semaphorins are involved in axonal guidance and are key regulators of the motility and morphology of the neurons [32].

Overall, WGCNA uncovered clusters of co-expressed proteins enriched in some terms directly related to neuronal functions (M1, M3, and M5). In addition, WGCNA modules (M1, M5) showed a consistent pattern of GO and pathway enrichments that was also detected in TopDE with a consistent directionality for ASD cases versus HCs. For instance, SNARE vesicular transport and ErbB signaling pathway were upregulated in ASD cases; semaphorins were downregulated in ASD cases. For each module, the eigenprotein and its hub proteins are plotted in Figure 4d. Enrichment of immune-related function was evidenced in all the significant modules and the TopDE list; however, it largely dominated the red M7 module.

### 2.6. SNARE Vesicular Transport Pathway and Axon Regeneration Proteins Correlate with ASD Severity

**Figure 5:**
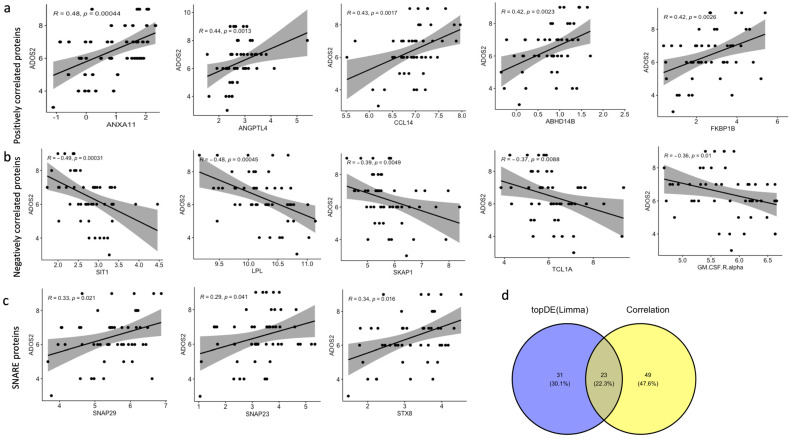
Correlation analysis between the protein NPX values and ADOS-2 score. (**a**) The top five positively correlated proteins with the ADOS-2 score. (**b**) The top five negatively correlated proteins with the ADOS-2 score. (**c**) The correlation between SNARE vesicular transport proteins and the ADOS-2 score. The correlation coefficients with the associated p-values are shown in each figure. (**d**) A Venn diagram of the overlapping proteins between the TopDE and the significantly correlated proteins (*p*-value < 0.05) with a correlation coefficient > 0.3 and < −0.3.

To identify proteins that correlate with ASD severity, we assessed the correlation between the ADOS-2 score, a tool for evaluating ASD diagnosis and severity across different ages and developmental stages, with the expression level (NPX values) of the proteins. The results (presented in Table S4a) showed that 64 proteins had a significantly moderate positive correlation and 7 proteins had a significantly moderate negative correlation with the ADOS-2 score. Eight proteins showed a correlation coefficient ≥0.4, namely FKBP1B, ANXA11, HMOX2, CD40, ANGPTL4, CCL14, ABHD14B, and COL18A1, and the first four were significantly upregulated in ASD cases (Figure 5a,b shows the top positive and negative correlated proteins). Subsequently, GO analysis was performed on the proteins with a significant *p*-value and a correlation coefficient of ≥0.3 or ≤−0.3 in order to recognize steady and relevant pathways between the TopDE and the significantly correlated proteins. GO enrichment analysis revealed an overrepresentation of the SNARE vesicular transport pathway in the KEGG database. Interestingly, all three proteins involved in the SNARE pathway (SNAP29, SNAP23, and STX8) had a significant yet weak to moderate positive correlation with the ADOS-2 scores and a correlation coefficient in the range of 0.29 to 0.34. The same three proteins were listed in the TopDE protein list (FC ≥ 2, BH-adjusted *p*-value < 0.05) (Table S1a). Therefore, SNARE vesicular transport was found to be enriched in GO of both the TopDE protein list and the ADOS-2-correlated protein list. Scatter plots of the correlations are shown in Figure 5c.

Furthermore, axon and neuron projection regeneration processes were enriched, and they included DAG1, SCARF1, FKBP1B, TN-R, ARHGAP1, PRDX5, TOP2B, and USP8 (Table S4), four of which (FKBP1B, ARHGAP1, PRDX5, and TOP2B) are listed in the TopDE protein list. On the other hand, the negatively correlated proteins, although less in number compared to the positively correlated ones, showed an overrepresentation of the extracellular region and the SH-2 domain (SKAP1 and SIT1), which is crucial for the protein kinase signaling cascade as it binds tyrosine-phosphorylated sequences in the proteins. In addition, the ADOS-2 scores were used to sub-group ASD individuals into three groups based on the severity levels (severe, moderate, and mild), and then the correlation was assessed using these three categories; the results are presented in Table S4b.

## 3. Discussion

Blood-based biomarkers are urgently needed to objectively diagnose and better understand the pathophysiological mechanisms behind ASD. Our proteomics study offers a deep insight into the blood proteome of Qatari ASD individuals using multiple computational methods, as this study is the first of its kind to comprehensively measure 1196 blood proteins using all the target panels developed by Olink (Uppsala, Sweden). We observed an overall dysregulation of the blood proteome between ASD cases and HCs with more upregulated proteins in ASD (237) compared to their 14 downregulated counterparts. In addition, we identified 15 proteins, summarized in Table 2, that could be potential biomarkers for ASD diagnosis, although further validation studies are needed. These proteins gave a high AUC, accuracy, and sensitivity and a lower specificity, which might be attributed to the small sample size and the heterogeneity of ASD. Some of the differentially expressed proteins identified in this cohort have been previously reported. Two of our upregulated proteins (uPAR and ARSB) and one of the downregulated ones (PTN) have been found to be differentially expressed in the serum of ASD cases using the SomaLogic platform with a similar directionality [24]. Moreover, another study that tested the correlation between serum and CSF proteins using three Olink panels (inflammation, cardiovascular I, and oncology I) found a total of 32 proteins that were associated with ASD diagnosis or autism score using a social responsiveness scale. Five of these proteins (AXIN1, NEMO, CD244, CASP8, DKK1, and SIRT2) were significantly expressed in our cohort [33]. In addition, IL-8 was found to be significantly upregulated in another study using the MesoScale Discovery platform, which is consistent with our findings [34]. Similarly, another study conducted on the postmortem brain of ASD showed upregulation of proinflammatory response markers IL-6, TNF-alpha, IL-8, and INF-Y [35], and the latter three markers were also significantly upregulated in our cohort. It is noteworthy that our study found global changes in immune-related markers, which is supported by many proteomics and transcriptomics studies in both the blood and brain tissue [35,36,37,38]. In addition, although ASD is a very heterogeneous condition, this study identified convergent mechanisms between ASD subjects by applying multiple approaches that differ in their essences, such as differential expression analysis, WGCNA, and correlation analysis. These approaches allowed us to identify redundant pathways that seemed to be enriched and may be of particular importance for ASD. Herein, we will discuss these pathways and their relevance to ASD.

We demonstrated that the ErbB (epidermal growth factor receptor signaling family) pathway was enriched in ASD cases compared to HCs (Figure S2a). ErbB belongs to the receptor tyrosine kinase family, which is important for cell proliferation, differentiation, growth, and migration [39]. Our study revealed that three ligands of this pathway (EGF, EREG, and TNF-alpha) were significantly upregulated in ASD cases; two of these ligands (EGF and EREG) were also found in the diagnostic biomarker list (Panel A). EGF plays a crucial role in the growth of the midbrain during embryogenesis, and it enhances dopamine uptake and dopaminergic neuron survival [40]. A study that measured EGF in the serum of ASD subjects showed that it is significantly elevated in ASD compared to healthy controls [41], which is consistent with our findings. Another study subcutaneously administrated EGF in neonatal mice, and this caused a range of neurological changes that included a reduction in social interaction and motor activity [42]. Although EGF did not correlate with the ADOS-2 score in our cohort, EREG did show a moderate positive correlation. Even though EREG has not been reported previously in the context of ASD, a study showed that overexpression of EREG in the brain could lead to a brain tumor, and it was involved in tumor exacerbation in a glioblastoma cell line [43]. Interestingly, TNF-alpha, which is another ligand for the ErbB signaling pathway, was elevated in the postmortem brain and serum of ASD cases compared to HCs [35,41]. TNF-alpha has a crucial role in initiating inflammation and maintaining tolerance to self-antigens by controlling lymphocyte survival and proliferation [44]. Furthermore, mutation and upregulation of the ErbB signaling receptors (i.e., EGFR) have been associated with different types of cancers, such as breast and colon cancer [45,46]. Thus, the role of the ErbB signaling pathway and its ligands (EGF, EREG, and TNF-alpha) needs further investigation in the context of ASD.

The second pathway highlighted by the proteomic profiling is the SNARE vesicular transport pathway. Three proteins of the SNARE vesicular transport pathway (SNAP29, SNAP23, and STX8) were significantly upregulated in ASD cases compared to the HCs (Figure S2b). In addition, these proteins showed a moderate correlation with ASD severity score and were co-expressed in the same module in WGCNA. In vertebrates, SNAP29 and SNAP23 belong to the SNAP25 protein subfamily. SNAP23 and SNAP25 proteins regulate exocytosis and can compensate for each other according to a cross-rescue experiment, as SNAP23 overexpression can rescue SNAP25 function in a SNAP25 KO model [47]. Although SNAP25 is almost exclusively expressed in neurons, SNAP23 can be expressed in neurons (neuronal cell body, somatodendritic compartment, and astrocytes) and other tissues of the body [48]. In addition, SNAP23 is localized in the plasma membrane and predominantly expressed in the post-synapse. Given its importance in a wide range of cell types, SNAP23 gene deletion leads to embryonic lethality in mice [49]. On the other hand, SNAP29 is prominently localized in the endosomal system and Golgi and is expressed at lower levels in the synaptic vesicles [50]. Overexpression of SNAP29 in a SNAP25 KO model showed that SNAP29 compensated for SNAP25 function; however, it caused a slower synaptic release and resulted in smaller neurons with fewer synapses [47]. Genetic studies have linked a specific allele polymorphism and copy number variants of SNAP29 to neuropsychiatric disorders such as schizophrenia [51]. SNAP25 subfamily proteins interact with a broad spectrum of syntaxin (STX) proteins to facilitate endosomal fusion [48]. In our cohort, STX8 was in the TopDE protein list. Mutations in SNARopathy genes can lead to diverse neurodevelopmental, cognitive, and motor delays and autistic traits [52].

It is important to mention that for 18 proteins differentially expressed in our cohort, their corresponding genes have been associated with ASD according to the Simons Foundation Autism Research Initiative (SFARI) genetic database. Two of these are from the TopDE list. The first is TOP2B (FC = 2.9). This gene encodes DNA topoisomerase protein, an enzyme that controls the topological landscape of the DNA during transcription; in addition, it catalyzes the breaking and rejoining of the two-strand DNA fragments [53]. According to SFARI, TOP2B was identified as a candidate gene for ASD as it has been reported to involve de novo mutations. It has been shown that knocking down TOP2B resulted in significantly reducing the transcription rate of long genes in neurons [54]. The ramification of TOB2P upregulation needs to be studied in the context of ASD. The second protein is PLXNA4 (FC = 2.4). This protein plays an essential role in mediating semaphorin signals and thus mediating axonal guidance during development [55]. Furthermore, copy number variants have been reported to encompass PLXNA4 in two unrelated cases of ASD [56].

It should be noted that this study was limited by the small sample size, particularly in the HC group, in addition to the male-to-female ratio imbalance between the ASD cases and HCs. The biomarkers identified in the present study showed a high AUC, accuracy, and sensitivity; however, the specificity was low, which might be caused by the small sample size and ASD heterogeneity. This sheds light on the imperative need for the stratification of ASD individuals based on their multi-omics profile, as this may help in identifying diagnostic biomarkers with high performance characteristics [57]. In addition, a validation cohort is needed to confirm our findings. Performing functional studies using animal models, organoids, and neuronal cell culture is essential to confirm and understand the pathophysiological role of these proteins in ASD in different dimensions. Finally, since our cohort was formed from the Qatari population, part of our future plans is to validate our findings using other populations.

## 4. Materials and Methods

### 4.1. Study Cohort

The study was performed according to the guidelines of the Declaration of Helsinki and was ethically approved (QBRI-IRB 2018-024). Written informed consent and assent were given by both parents and children, respectively, for all ASD individuals and healthy controls (HCs). The HCs’ communication and social skills were evaluated using the Social Communication Questionnaire (SCQ). ASD individuals were clinically diagnosed using the *Diagnostic and Statistical Manual of Mental Disorders, Fifth Edition* (DSM-5), and their severity levels were assessed using the second edition of the Autism Diagnostic Observation Schedule (ADOS-2) test. Demographical information was also obtained from the participants during the visit and is summarized in Table 1.

### 4.2. Olink Proteomics Analysis and Data Pre-Processing

Human serum samples were collected, processed, and stored (frozen at −80 °C) at Qatar Biomedical Research Institute (QBRI), Qatar. Differential protein discovery was performed using the Olink platform (Uppsala, Sweden), which utilizes proximity extension assay (PEA) technology that combines dual recognition of proteins by pairs of antibodies labeled with a DNA oligonucleotide; when bound in close proximity, the dual antibody recognition allows the hybridization of the DNA probes. This step is followed by DNA amplification for each corresponding protein signal. The signals are in the form of cycle threshold (Ct). The Ct signals were then normalized and reported as normalized protein expression (NPX) values in a log2 scale, which is an arbitrary unit for the relative quantification of proteins across samples. Prior to running the panels, the serum samples were randomized and aliquoted in 96-well plates. The serum samples were analyzed using all 13 Olink target panels (neurology, development, neuro-exploratory, inflammation, immune response, cell regulation, organ damage, metabolism, oncology II, oncology III, cardiometabolic, cardiovascular II, cardiovascular III) available from Olink (Uppsala, Sweden). These panels combined cover a total of 1196 measured proteins. Olink’s standard protocol was followed, and the runs were performed at QBRI in an Olink-certified proteomics core facility. Samples that passed the QC assessment were used for downstream analysis. In addition, proteins were excluded from the analysis if ≥30% of cases and controls were below the lower limit of detection (LLOD). Missing values were imputed using missForest package in R. Sample outliers were identified using principal component analysis (PCA) and hierarchal clustering; then they were excluded from the analysis as illustrated in Figure S3.

### 4.3. Statistical Analysis

Differentially expressed proteins were identified using the *Limma* (Linear Models for Microarray Data) package in R. The *p* -values of all the proteins were adjusted for multiple testing using the Benjamini–Hochberg (BH) method. In addition, age and sex were considered as covariates in the model. Top differentially expressed proteins (TopDE) were selected based on the fold change (FC ≥ 2) and BH-adjusted *p*-value (adj *p*-value ≤ 0.05). Correlation analysis was performed using cor.test() function, and proteins that exhibited a correlation coefficient of ≥0.3 or ≤−0.3 and a *p*-value < 0.05 were considered for further enrichment analysis. Furthermore, the *t*-test() function was used to test the significant difference in eigenprotein between ASD cases and HCs in the important WGCNA modules. The heatmap was constructed using Heatmap() function from the ComplexHeatmap package. ROC curve analysis was performed using the pROC package. All analyses were conducted in R Studio (version 1.3.1093).

### 4.4. Weighted Gene Co-Expression Analysis

Signed co-expression networks were built using the WGCNA package in R as described [30]. Weighted gene (protein) co-expression analysis is a method for describing the correlation patterns among genes (proteins) and for finding clusters (modules) of highly correlated genes (proteins). The generated clusters are summarized in the form of the eigengene (eigenprotein), which is the first principal component of each sample’s expression data. A similarity correlation matrix was produced from all the proteins; then, an adjacency matrix was calculated using a soft-threshold power of 4, chosen based on the scale-free topology criteria. Subsequently, a topological overlap matrix (TOM) was constructed from the adjacency matrix to build the modules. The hierarchical cluster was built using the topological overlap dissimilarity (dissTOM). Then, the modules were depicted in the clustering tree. The module hierarchical cluster cut was performed at 0.35 to merge the closely related modules. A minimum of 30 proteins per module was set to generate a moderately large module size. Proteins that were not assigned to any modules were listed in the grey module.

### 4.5. Protein Enrichment Analysis

For enrichment analysis, g:Profiler (https://biit.cs.ut.ee/gprofiler (accessed on 27 November 2022)), an online web-server that performs statistical enrichment analysis using the p:GOSt function, was used with the default setting to detect overrepresented terms in Gene Ontology (GO), such as cellular components (CCs), molecular functions (MFs), and biological processes (BPs), as well as overrepresented pathways in KEGG and REACTOME databases. All the significantly enriched terms and their associated proteins were selected based on the Fisher exact test (FET) with an adjusted *p*-value < 0.05. The hub proteins were identified by extracting the proteins from each WGCNA module. Next, the proteins were plugged into STRING (version 11.5) to generate the interaction network files. To find the hub proteins, the extracted files were uploaded into CytoScape (version 3.9.1).

### 4.6. Feature Selection and ROC Curve Analysis

To identify a minimal set of biomarkers, we applied two feature selection methods. The first is the minimal unbiased variable selection (MUVR) algorithm, a machine learning technique that aims to identify the minimal set of variables that are required for the successful classification of a given dataset [27], and it was applied to the list of top differentially expressed proteins (TopDE). This algorithm employs a variety of techniques, including recursive variable elimination and repeated cross-validation, which help to minimize overfitting and improve the performance of the resulting model. Random forest (RF) was chosen, along with 21 outer and 28 inner cross-validation segments, and a variable ratio of 0.75. The list of minimal optimal subsets was selected.

The second method is Boruta [28], another feature selection method based on RF. Using statistical testing, it iteratively removes the features which are less relevant than randomized features. Boruta was also applied to the TopDE list, and the algorithm was run with maxRun = 1000 and all other parameters set to their default values.

To ensure the robustness of our selected variable set, further validation was performed using a variety of classification methods such as RF, support vector machines (SVMs), and generalized linear models (GLMs). We used the mlr3 (Machine Learning in R, version 3) framework to implement the classification methods. This framework provides a consistent interface for a wide range of machine learning tasks, including classification, regression, and clustering [58]. In addition, it ensures the reliability of the results. A four-fold cross-validation repeated 250 times was used on the dataset. Various performance evaluation measures were used to assess the performance of the model: accuracy, true positive rate (sensitivity), true negative rate (specificity), and the area under the receiver operating characteristic curve (AUC).

## 5. Conclusions

Overall, this study measured more than 1100 blood proteins of ASD using PEA technology. Our differential expression analysis revealed a difference in blood proteome profile between ASD cases and HCs. In addition, 15 proteins were selected by machine learning as potential diagnostic biomarkers for ASD. Our study identified two pathways that were upregulated in ASD (SNARE vesicular transport and ErbB signaling pathway). These pathways were enriched in the ASD blood proteome profile despite the high heterogeneity of ASD. Therefore, they might play an essential role as common underlying pathophysiological mechanisms in ASD. A validation cohort should be tested to confirm these findings, and patient stratification using a multi-omics approach may be the way forward toward better understanding and characterizing ASD.

## Figures and Tables

**Table 1:** Participants’ demographical information summary.

	ASD Cases	Healthy Controls
Number of participants	N = 91	N = 30
Age (Mean ± SD)	8.32 ± 2.29	11.1 ± 2.2
Gender (F/M)	19/72	15/15
ADOS-2 scores (Mean ± SD)	6.39 ± 1.47	-

ASD: autism spectrum disorder; ADOS-2: Autism Diagnostic Observation Schedule second edition; SD: standard deviation.

**Table 2:** MUVR and Boruta overlapping proteins, Panel A, that best discriminative between ASD and HCs.

Rank	Protein Symbol	Protein Full Name	MUVR Score *	Fold Change (FC)	Adjusted *p*-Value
1	TNFSF14	Tumor Necrosis Factor Ligand Superfamily Member 14	2.01	↑ 2.70	3.82 × 10^−8^
2	EGF	Epidermal Growth Factor	3.81	↑ 7.20	2.86 × 10^−11^
3	LAP.TGF.beta.1	Transforming Growth Factor Beta-1	9.44	↑ 2.05	3.82 × 10^−8^
4	JAM.A	Junctional Adhesion Molecule A	11.3	↑ 2.05	1.55 × 10^−8^
5	CD40.L	CD40 Ligand	15.58	↑ 3.50	2.9 × 10^−8^
6	GP6	Glycoprotein VI Platelet	21.32	↑ 2.7	1.55 × 10^−8^
7	ARHGAP25	Rho GTPase Activating Protein 25	27.05	↑ 2.30	1.58 × 10^−4^
8	CLEC1B	C-Type Lectin Domain Family 1 Member B	29.63	↑ 2.13	1.76 × 10^−8^
9	EREG	Epiregulin	30.27	↑ 5.06	2.03 × 10^−7^
10	ST1A1	Sulfotransferase Family 1A Member 1	40.72	↑ 3.87	2.37 × 10^−6^
11	ARSB	Arylsulfatase B	41.38	↑ 2.03	6.77 × 10^−7^
12	CASP2	Caspase 2	42.42	↑ 2.03	2.4 × 10^−7^
13	LSP1	Lymphocyte Specific Protein 1	42.57	↑ 2.27	2.05 × 10^−5^
14	MANF	Mesencephalic Astrocyte Derived Neurotrophic Factor	43.39	↑ 2.06	2.4 × 10^−7^
15	PTPN1	Protein Tyrosine Phosphatase Non-Receptor Type 1	43.43	↑ 3.20	7.9 × 10^−7^

* MUVR scores represent the average ranks over all the individual cross-validations performed (21 outer and 28 inner cross-validation segments).

## Data Availability

The data presented in this study are available on request from the corresponding author.

## Supplementary Materials

The following supporting information can be downloaded at: https://www.mdpi.com/article/10.3390/ijms24087443/s1.
