# Supplementary material for: Blood Proteomics Analysis Reveals Potential Biomarkers and Convergent Dysregulated Pathways in Autism Spectrum Disorder: A Pilot Study
Source: Int J Mol Sci. 2023 Apr 18;24(8):7443. doi: 10.3390/ijms24087443 (PMC10138652; doi:10.3390/ijms24087443)
Supplement: Supplementary file 1 [file ijms-24-07443-s001.zip › Figure.S1.pdf]

a

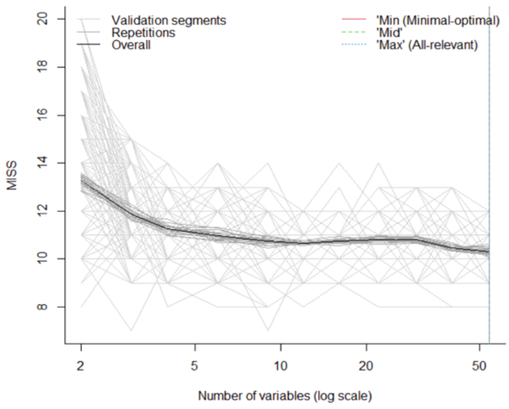

b

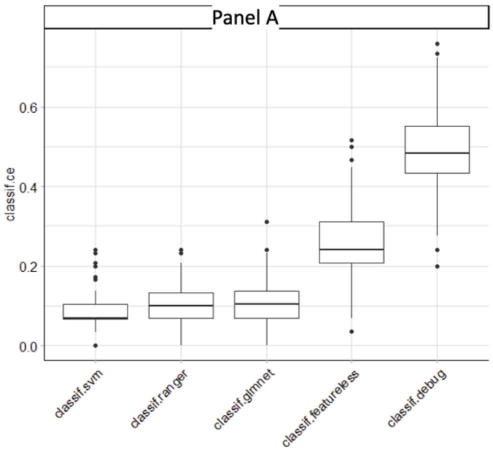

c

Panel A	SVM	RF	Glmnet	featureless	debug
AUC, (95% CI)	0.876, (0.697-1.00)	0.868, (0.698-1.00)	0.913, (0.794-1.00)	0.50, (0.50)	0.505, (0.237-0.773)
Accuracy%, (95% CI)	91.4%, (82.7-100%)	90.7%,(81.9-99.5%)	88.4%, (79.2-97.5%)	74.3%, (60.7-87.9%)	50.4%, (31.9-68.9%)
Sensitivity%, (95% CI)	99.9%, (99.7-100%)	99.2%, (95.5-100%)	95.8%, (86.1-100%)	100%, (100%)	50.4%, (29.7-71.1%)
Specificity%, (95% CI)	66.2%, (33.8-98.6%)	65.7%, (32.8-98.6%)	67.2%, (34.2-100%)	0.0%, (0.0%)	50.6%, (11.8-89.4%)

Figure S1. Machine learning approach for feature selection and classification. (a) The misclassification rate as a function of the number of included variables. The light grey lines in the graph represent the validation performance of individual inner segments, while the darker grey lines show the average validation performance of all inner segments. The minimal-optimal ("Min") and all-relevant ("Max") models represent the two extremes of variable selection, with validation performance (measured in misclassifications) minimized within 5% of the minimum. The minimal-optimal model represents the minimum number of variables needed for optimal method performance, such as in biomarker discovery, while the all-relevant model includes all variables with relevant signal-to-noise for the research question, including redundant but non-erroneous variables. The "Mid" model represents a compromise between the "Min" and "Max" models, found at the geometric mean. **(b)** A Comparison of classification error rates for five different classification algorithms: SVM, RF, GLMnet, Featureless, and Random Guessing on Panel A (the overlapping proteins between MUVR and Boruta). The Featureless algorithm assigns all samples to the largest class, while the Random Guessing algorithm randomly assigns samples to classes. These algorithms are included for comparison. The SVM algorithm showed the best performance, with error rates similar to those of the Random Forests and GLMnet algorithms and much better than Featureless and Random Guessing algorithms. **(c)** The performance characteristics of different classification algorithms.
