# Supplementary material for: Blood Proteomics Analysis Reveals Potential Biomarkers and Convergent Dysregulated Pathways in Autism Spectrum Disorder: A Pilot Study
Source: Int J Mol Sci. 2023 Apr 18;24(8):7443. doi: 10.3390/ijms24087443 (PMC10138652; doi:10.3390/ijms24087443)
Supplement: Supplementary file 1 [file ijms-24-07443-s001.zip › Figure.S2.pdf]

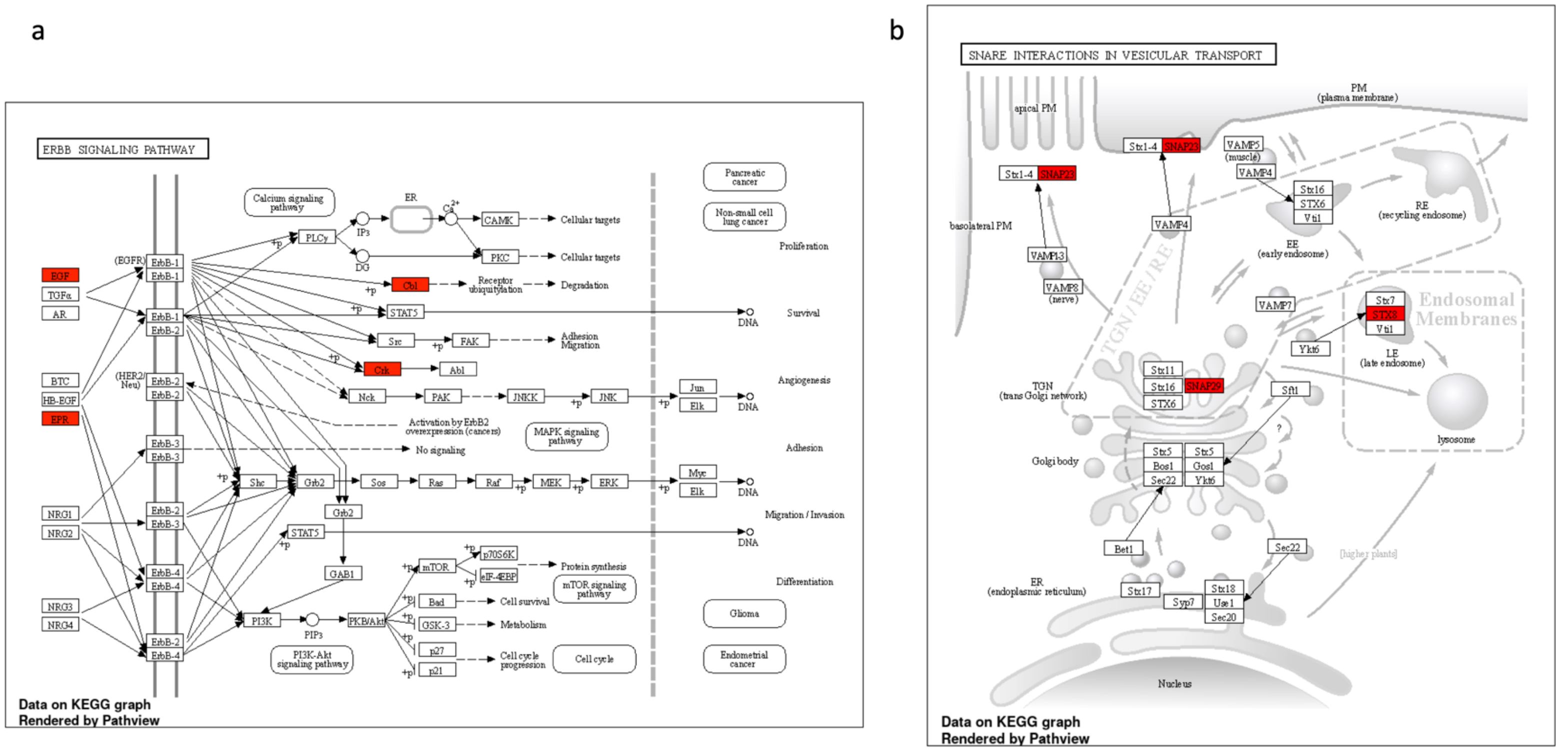

Figure S2. ASD dysregulated pathways. (a) ErbB signaling pathway. **(b)** SNARE interaction in vesicular transport. In red are the proteins that were differentially expressed (up-regulated).
