# Supplementary material for: Blood Proteomics Analysis Reveals Potential Biomarkers and Convergent Dysregulated Pathways in Autism Spectrum Disorder: A Pilot Study
Source: Int J Mol Sci. 2023 Apr 18;24(8):7443. doi: 10.3390/ijms24087443 (PMC10138652; doi:10.3390/ijms24087443)
Supplement: Supplementary file 1 [file ijms-24-07443-s001.zip › Figure.S3.pdf]

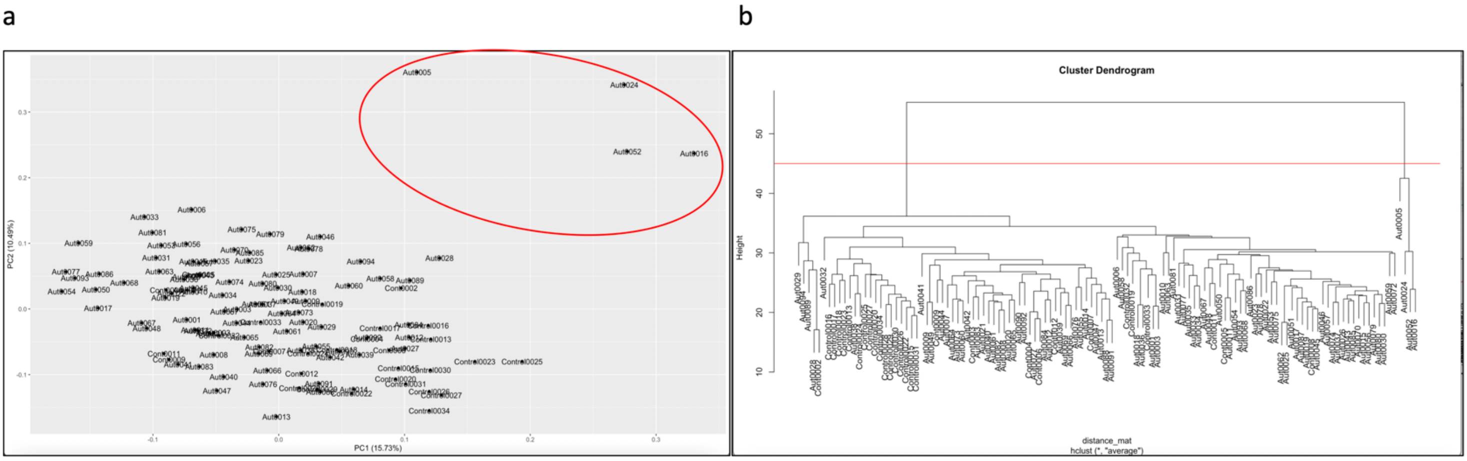

Figure S3. Sample outlier identification. (a) A PCA plot of all the individual samples using PC1 (x-axis) and PC2 (y-axis), the red circle highlights the sample outliers. **(b)** Hierarchical clustering of all the samples, the red line represents the sample outliers' cutoff value.
